# Pelvic Mass 21 Years after Total Hip Arthroplasty

**DOI:** 10.1155/2013/424319

**Published:** 2013-06-20

**Authors:** Joe Miller, Thomas H. Tarter

**Affiliations:** ^1^Department of Urology, University of California San Francisco, San Francisco, CA, USA; ^2^Cancer Care Center of Decatur, Decatur, IL, USA

## Abstract

*Background*. Long-term urologic complications after total hip arthroplasty are rare, and reports in the urologic literature are scant. We present a recent case and review the relevant literature. *Case*. A 77-year-old man was referred to the urology clinic for a single episode of gross painless hematuria, abnormal urine cytology, and pelvic mass. He had a significant smoking history. Surgical history included right total hip arthroplasty 21 years prior. *Results*. Pelvic ultrasound revealed a large mass abutting the right bladder wall. Subsequent computed tomography indicated that the mass was extrinsic to the bladder. Results of computed tomography-guided biopsy of the mass were consistent with foreign body granuloma. Surveillance imaging confirmed no growth or progression, and intervention was deferred. *Conclusion*. Long-term complications of total hip arthroplasty may present with signs and symptoms of urologic disease. Reports in the urologic literature are rare.

## 1. Introduction

Urinary retention and urinary tract infection are common postoperative complications after total hip arthroplasty (THA) but long-term urologic complications are rare. We report a case of a patient with a history of THA referred for hematuria, abnormal urine cytology, and a pelvic mass and present a review of the relevant literature.

## 2. Case Report

A 77-year-old man was referred to the urology clinic for evaluation of a single episode of gross painless hematuria, abnormal urine cytology, and pelvic mass. The patient had a previous 30 pack-year smoking history but had quit 12 years prior. His past medical history was significant for hypertension, hyperlipidemia, and gout. His past surgical history included right THA 21 years prior, with revision 10 years prior to referral.

**Figure 1:**
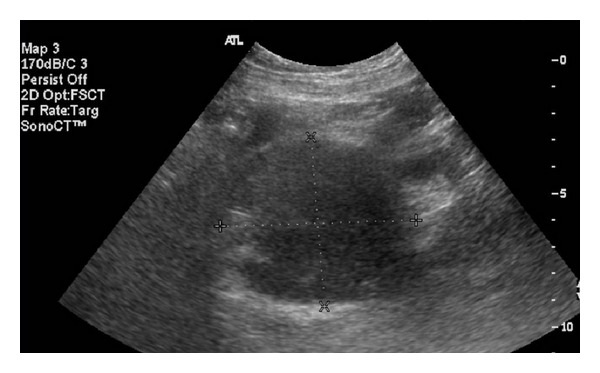
Pelvic ultrasound showing large mass adjacent to right bladder wall.

**Figure 2:**
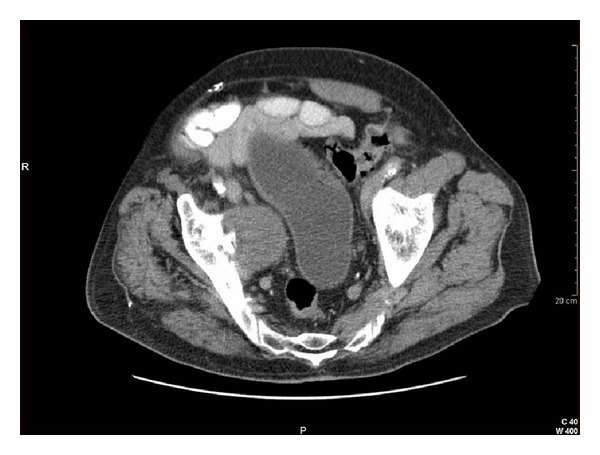
CT scan of pelvis revealing large mass to be extrinsic to bladder wall.

On presentation, he denied abdominal or pelvic pain, dysuria, or further episodes of gross hematuria. Physical exam was normal. Repeat voided urine cytology was negative for malignant cells. Pelvic ultrasound showed an extrinsic mass adjacent to the right bladder wall (Figure 1). Review of the subsequent computed tomography (CT) of the abdomen and pelvis revealed a 5.7 × 4.6 cm mass in the right pelvis causing mass-effect distortion of the bladder. No invasion of the bladder wall was evident (Figure 2). A CT-guided biopsy of the mass produced only acellular material. Cystoscopic examination revealed no evidence of urothelial tumor or lesion or foreign body intrusion. A follow-up CT performed at an interval of 3 months showed no change in the size or character of the mass, and observation was discontinued, with planned intervention only in the case of clinical worsening.

## 3. Discussion

The incidence of significant urologic complications after THA is low. In the immediate postoperative period, urinary retention and urinary tract infection occur in as many as 25–35% of patients [1, 2]. Long-term urologic complications including hematuria, fistula, and pelvic mass have been reported, but are rare.

Several cases of hematuria following THA have been reported. Four cases of hematuria occurring 7–14 days postoperatively were attributed to necrosis of the bladder wall by the exothermic polymerization of the methacrylate cement [3]. A case of delayed hematuria secondary to cement intrusion into the bladder occurring at 1 year postoperatively was reported by Memon et al. [4]. Erosion of the bladder wall by prosthesis components, screws, or cement and the subsequent development of vesicoacetabular or vesicocutaneous fistula have been documented as late as 23 years after THA [5–7]. Medial migration of components compromising the bladder or ureter has also been reported, including a case of complete acetabular component migration into the bladder [8].

Migration of polyethylene debris and subsequent formation of a solid or cystic mass have been reported in both the orthopedic and gynecologic literature. In one such case a woman was referred to the department of gynecology with a 5-year history of an enlarging, palpable pelvic mass. CT of the pelvis revealed a 20 × 16 × 12 cm mass compressing sigmoid colon and bladder and causing complete obstruction of the left ureter. Histopathologic examination of the excised mass revealed fibrous necrotic tissue and polyethylene wear particles [9]. Mak et al. presented a case of a woman who underwent surgical excision of an “ovarian tumor” which proved to be one-half of a large bilobar cyst containing metal debris and fragments of polyethylene [10]. In a similar report, an elderly woman with invasive breast cancer was found to have 12 × 11 × 10 cm pelvic mass which appeared to arise from the right adnexa. An exploratory laparotomy performed immediately following total mastectomy revealed a retroperitoneal mass containing necrotic debris and foreign-body material. After the debris was removed and the area irrigated, screws and cement were found emanating from the pelvic sidewall. No evidence of metastatic disease was found on histopathologic examination of the contents of the pelvic mass [11].

Recent reports of complications after THA in the urologic literature are scant. In 1979, Ray et al. published the case report of a woman with extruded cement displacing her bladder and ureter, and in 1980, Solomon and MacGregor reported formation of an urethrocutaneous fistula following THA [12, 13].

## 4. Conclusion

Long-term urologic complications following THA are rare. Pelvic mass after THA has been widely reported in the orthopedic literature. Isolated reports exist in the gynecologic and radiologic literature, but there appears to be little awareness in the urologic community. Patients with a pelvic mass after THA who are referred for urologic complaints present a diagnostic dilemma.
